# Perfusion index for assessing microvascular reactivity in septic shock after fluid resuscitation

**DOI:** 10.5935/0103-507X.20180027

**Published:** 2018

**Authors:** Igor Alexandre Côrtes Menezes, Cláudio Leinig Pereira da Cunha, Hipólito Carraro Júnior, Alain Marcio Luy

**Affiliations:** 1 Unidade de Terapia Intensiva, Hospital de Clínicas, Universidade Federal do Paraná - Curitiba (PR), Brasil.; 2 Departamento de Clínica Médica, Hospital de Clínicas, Universidade Federal do Paraná - Curitiba (PR), Brasil.

**Keywords:** Perfusion index, Septic shock, Hyperemia, Microcirculation

## Abstract

**Objective:**

Microcirculation disturbances are implicated in the prognosis of septic shock. Microvascular hyporesponsiveness can be assessed by an oximetry-derived perfusion index and reactive hyperemia. Using this perfusion index, we investigated reactive hyperemia and its relationship with peripheral perfusion and clinical-hemodynamic parameters in septic shock.

**Methods:**

Eighty-two patients were evaluated: 47 with septic shock and 35 controls. Tests were performed within 24 hours after admission. The perfusion index was evaluated before and after a 3-min blood flow occlusion using a time-response analysis for 5 min. The perfusion index was also evaluated in the hyperemic phases and was mainly derived by mechanosensitive (ΔPI_0-60_) and metabolic mechanisms (ΔPI_60-120_). Correlation tests were performed between reactive hyperemia and clinical-hemodynamic data.

**Results:**

Reactive hyperemia measured by the perfusion index was significantly lower in patients with septic shock, but this was only observed for the first 45 seconds after cuff-deflation. In the remaining period, there were no statistical differences between the groups. The peaks in the perfusion index were similar between groups, although the peak was reached more slowly in the septic group. Values of ΔPI_0-60_ were lower in shock [01% (-19% - -40%) *versus* 39% (6% - 75%); p = 0.001]. However, ΔPI_60-120_ was similar between the groups [43% (18% - 93%) *versus* 48% (18% - 98%); p = 0.58]. The time-to-peak of the perfusion index was correlated positively with the SOFA scores and negatively with C-reactive protein; the peak of the perfusion index was positively correlated with vasopressor doses; and the ΔPI_60-120_ values were positively correlated with C-reactive protein and vasopressor doses. No other significant correlations occurred.

**Conclusions:**

This perfusion index-based study suggests that septic shock promotes initial peripheral vascular hyporesponsiveness and preserves posterior vascular reactivity to a considerable degree. These results demonstrate a time-dependent peripheral hyperemic response and a significant ischemic reserve in septic shock.

## INTRODUCTION

Septic shock, i.e., circulatory failure due to sepsis that leads to tissue hypoperfusion, is still a clinical syndrome with high mortality.^(^^1^^)^ Microvascular disturbances have been identified in human sepsis, even when systemic hemodynamics have been corrected,^(^^2^^)^ and their severity is related to outcomes.^(^^2^^,^^3^^)^ Among the main disturbances are endothelial dysfunction and vascular hyporesponsiveness.^(^^3^^,^^4^^)^ Vascular hyporesponsiveness is caused by multiple disruptions in cellular homeostasis and vascular damages.^(^^5^^)^ Clinical evidence strongly suggests that vascular hyporeactivity could contribute to peripheral hypoperfusion and the severity of organ failure in septic shock when evaluated in nitric oxide (NO)-dependent arteries and in skeletal muscle microcirculation.^(^^4^^,^^6^^)^

Nevertheless, when performed in the skin tissue, some results seem contradictory. In a study of critically ill patients (one-third with sepsis), the vascular reactivity was not different between different degrees of organ dysfunction.^(^^7^^)^ Another study showed that skin endothelial reactivity remains intact in septic shock.^(^^8^^)^ Finally, septic neonates showed an increased skin vascular reactivity compared to controls.^(^^9^^)^ Recently, there is an increasing interest in monitoring shock using non-vital vascular beds to assess skin circulation.^(^^10^^,^^11^^)^ Thus, a better understanding of peripheral vascular reactivity in septic shock is needed.

Post-occlusive reactive hyperemia, a known vascular reactivity test, refers to an increase in organ blood flow above the baseline levels following release from brief arterial occlusion. This flow increase estimates the vascular response to the maximal demand of tissue, making this test attractive for monitoring hypoperfusion states as septic shock.^(^^4^^,^^12^^)^ Multiple and organ-dependent mechanisms are involved, including NO bioavailability, sensory nerves, myogenic reflex, hyperpolarizing factors and cyclooxygenase metabolites.^(^^12^^)^

New oximeters can calculate the perfusion index (PI) from a pulsatile photoplethysmography signal, mostly of skin microcirculation,^(^^13^^)^ and indirectly measure the perfusion variations.^(^^14^^)^ Hypoperfusion measured with PI has been shown to be predictive of mortality in sepsis,^(^^15^^)^ and recent reports have shown that reactive hyperemia can be evaluated using PI.^(^^16^^,^^17^^)^

Therefore, this preliminary study used PI to evaluate the global vascular reactivity in septic shock using a time-response analysis of the reactive hyperemia. It also aimed to verify the relationship between this parameter, peripheral perfusion, systemic macro-hemodynamics, vasopressors doses and organ failure scores.

## METHODS

All participants provided written informed consent, and the research was approved by the Research Ethics Committee of the *Universidade Federal do Paraná* (protocol number: 685.344/2014). Therefore, the study protocol was in accordance with the national and international ethical norms on research with human beings (conforming to the Declaration of Helsinki).

This transversal observational study was conducted in the intensive care unit (ICU) at *Hospital de Clínicas* of the *Universidade Federal do Paraná* from September 2014 to April 2016. The study selected consecutive adult patients admitted with the diagnosis of septic shock or within 24 hours after septic shock onset in patients previously admitted for other causes.

According to internationally accepted consensus definitions at the time of the beginning of study,^(^^18^^,^^19^^)^ sepsis was defined based on clinical evidence of infection and 2 or more of the following: (1) fever axial temperature greater than 38°C or hypothermia (axial temperature < 36°C), (2) tachycardia (heart rate > 90 beats per minute), (3) tachypnea (> 20 breaths per minute) or need for mechanical ventilation, (4) leukocytosis (> 12,000 cells/mm^3^) or leukopenia (< 4,000 cells/mm^3^), or a ratio of greater than 10% band cells to polymorphonuclear cells. Septic shock was defined as sepsis with hypotension and/or hypoperfusion represented by acute hyperlactatemia (irrespective of blood pressure), even after initial volume expansion, and requiring vasopressors.

Exclusion criteria: Severe hepatopathy/coagulopathy, infective endocarditis, systemic sclerosis and severe obstructive arterial disease. These criteria were chosen to reduce the risks of possible hemorrhagic and ischemic complications of the procedure.

### Controls

Reactive hyperemia assessment using PI was also carried out in a convenience sample of subjects matched by age, sex and without signs of clinical infection or acute illness. Due to the importance of hypertension, diabetes mellitus and smoking to microvascular reactivity,^(^^17^^)^ the groups were approximately matched for these factors.

All patients received broad-spectrum antibiotic coverage. The local hemodynamic support was as follows: central venous pressure was 8 - 12mmHg, mean arterial pressure (MAP) was > 65mmHg, urine output was > 0.5mL/kg/hour, and central venous oxygen saturation (ScvO_2_) was > 70%.^(^^19^^,^^20^^)^ Initially, all patients received 30mL/kg crystalloid fluid over 1 hour. Fluid administration was continued until there was no response to passive-leg raising or no respiratory variations of the inferior vena cava diameter (Samsung Medison Ultrasound; Seoul, Korea). If MAP remained < 65mmHg after fluid administration, a diagnosis of septic shock was made, and norepinephrine was titrated to maintain MAP > 65mmHg. Intensivists were blinded to peripheral perfusion variables. The capillary refill time was also evaluated, and it was considered prolonged if it was longer than 4.5 seconds.

The information collected included demographic characteristics, diagnosis for admission and comorbidities, Acute Physiology and Chronic Health Evaluation II score (APACHE II) and Sequential Organ Failure Assessment (SOFA) score. Assessment of patients occurred within 24 hours after admission to the ICU with a diagnosis of shock or within 24 hours after the onset of shock in patients who were previously admitted for other causes. All hemodynamic, metabolic and PI variables were measured after fluid resuscitation.

Simultaneous blood gases from arterial and central venous catheters were obtained. Samples were taken in a 3mL heparinized syringe. Blood gas analysis and lactate concentration were determined using the GEM premier 3000 Gasometer (Barcelona, Spain). Central venous oxygen saturation was calculated from a sample from the central venous catheter. The central venous-arterial blood carbon dioxide partial pressure difference (Pv-aCO_2_) was calculated as the difference between the partial pressures of central venous carbon dioxide (PcvCO_2_) and arterial carbon dioxide (PaCO_2_). We also collected other data, including primary site, type of infection and cause of death.

### Reactive hyperemia measured with the perfusion index

The perfusion index was measured by attaching a pulse oximeter probe (Masimo Radical, Masimo-Corp. CA). The same researcher performed all tests. All studies were performed after resuscitation and at least 1 h of hemodynamic stability (no change in vasopressor dose or fluid boluses) in a controlled room (25°C). The pulse oximeter was placed on the index finger. The PI was measured for a period of 5 min (basal value) after signal stabilization. Subsequently, a sphygmomanometer cuff was inflated around the homolateral arm, 30 - 50mmHg above the systolic pressure, to occlude the arterial flow for a period of 3 min.^(^^4^^,^^6^^,^^17^^)^

Reactive hyperemia occurred on deflation of the cuff. The PI was determined every 15 seconds for a period of 5 min to create a curve of PI variation (ΔPI) as a function of time. The variation in the PI (Δ) was calculated at each assessed time point using the following formula:

**Table:** 

ΔPI: PI time - PI basal/PI basal (x 100)

The peak of PI (ΔPI peak) and time to reach the peak (time to peak) were also measured. Next, the mean of the delta of PI was determined between 0 and 60 seconds after cuff deflation (ΔPI_0-60_) and 60 to 120 seconds after cuff deflation (ΔPI_60-120_). These time intervals were specially chosen to evaluate the phases of reactive hyperemia, which were mainly generated by mechanosensitive mechanisms and metabolic factors, respectively.^(^^21^^)^ The ΔPI_0-60_ and ΔPI_60-120_ were compared between groups.

### Statistical analysis

The Shapiro-Wilk test was used to test normalcy of the sample. Nonparametric values were expressed as medians/interquartile ranges and categorical variables were expressed as percentages. The Mann-Whitney test and Chi-square test were used to determine the significance of the differences in the nonparametric and categorical variables, respectively. Spearman correlation analysis was conducted to determine the relationships between the reactive hyperemia and the clinical-hemodynamic parameters. The statistical program GraphPad Prism 3.02 was used for analyses. The significance level sought was p < 0.05. Because this was the first study in the literature to use this design to evaluate septic shock (time-response analysis using PI), the sample size was not exactly calculated. To minimize this issue, we observed the sample size selected by other similarly designed studies in the literature, which was between 15 and 42 patients per septic group and between 15 and 38 patients in control group.^(^^4^^,^^6^^,^^7^^)^

## RESULTS

There were 47 septic shock patients included. In-hospital mortality of septic shock was 46.8% (22/47). The clinical-demographic and hemodynamic data of the patients are listed in table 1. Taken as a whole, these data describe a heterogeneous critically ill population, typical of sepsis. The control group included 35 patients. There were no differences in age, sex, prevalence of hypertension, diabetes and smoking between the groups. The septic shock group showed a statistically significant increased heart rate, similar values of arterial pressure and reduced peripheral perfusion measured with the PI. In addition, there were 9 patients (19%) with prolonged capillary refill time in the septic group and no patients in control group.

**Table 1:** The demographic, clinical and basal perfusion index values of the control patients and the patients with septic shock after fluid resuscitation

Parameters	Control n = 35	Septic shock n = 47	p value
Clinical			
Age (years)	63 (57 - 67)	59 (45 - 74)	0.757
Sex (f/m)	17/18	22/25	0.874
Arterial hypertension n (%)	15 (42)	21 (46)	0.869
Diabetes mellitus	8 (23)	11 (23)	0.953
Smoking	5 (14)	8 (17)	0.737
APACHE II score	N/A	20 (14 - 23)	---
SOFA score	N/A	10 (8 - 12)	---
Source of infection n (%)			
Respiratory	0	15 (31)	---
Abdominal	0	24 (51)	---
Urinary	0	4 (9)	---
Others	0	4 (9)	---
Noradreline dose (µg/kg/min)	0	0.4 (0.3 - 0.8)	---
Vasopressin use n (%)	0	13 (28)	---
C-reactive protein (mg/dL)	N/A	26 (19 - 32)	---
Hemodynamic			
MAP (mmHg)	93 (74-97)	81 (74 - 93)	0.070
HR (bpm)	66 (61-76)	105 (95 - 117)	< 0.001*
ScvO_2_ (%)	N/A	74 (67 - 81)	---
Urine output (ml/kg/hour)	N/A	0.4 (0.1 - 0.9)	---
Pv-aCO_2_ (mmHg)	N/A	5 (4 - 8)	---
Arterial lactate (mmol/L)	N/A	1.8 (1.2 - 2.5)	---
Peripheral perfusion			
Basal PI	5.6 (2.3 - 9.3)	3.6 (0.9 - 5.7)	0.013*

APACHE II - Acute Physiology and Chronic Health Evaluation; SOFA - Sequential Organ Failure Assessment; N/A - not applicable or not available; MAP - mean arterial pressure; HR - heart rate; ScvO_2_ - central venous oxygen saturation; Pv-aCO_2_ - venous to arterial carbon dioxide difference; PI - perfusion index.

* statistically significant p value for control *versus* septic shock.

**Figure 1:**
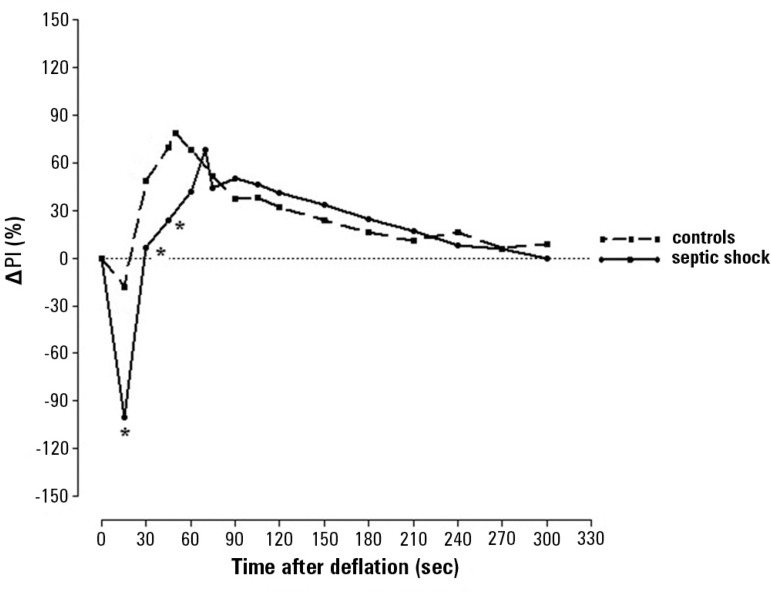
Delta of the perfusion index during reactive hyperemia in controls and patients with septic shock. The perfusion index was measured in fingertip microcirculation following forearm stagnant ischemia. Each point in the curves represents the median values for the groups at the evaluated time. ΔPI - Delta of perfusion index. * Statistically significant p value for septic shock *versus* control.

Figure 1 and table 2 shows the ∆PI after deflation of the sphygmomanometer cuff. There were evident and statistically significantly lower ∆PI values in the Septic shock group compared to controls at only 15, 30 and 45 seconds after cuff deflation. In the remaining evaluation period, there were no statistically significant differences between the groups.

**Table 2:** Delta of the perfusion index during reactive hyperemia in controls and patients with septic shock

Time after deflation (seconds)	ΔPI (%)		p value
	**Control** **n = 35**	**Septic shock** **n = 47**	
0	0	0	---
15	-19 (-56 - -9)	-100 (-100 - -43)	< 0.001*
30	48 (18 - 80)	7 (-16 - 31)	< 0.001*
45	70 (20 - 119)	25 (6 - 76)	0.010*
60	68 (25 - 116)	42 (14 - 95)	0.111
75	52 (21 - 112)	44 (20 - 97)	0.536
90	37 (18 - 98)	50 (16 - 92)	0.789
105	38 (12 - 80)	46 (16 - 97)	0.749
120	32 (8 - 71)	41 (14 - 79)	0.514
150	24 (2 - 68)	33 (7 - 51)	0.459
180	16 (1 - 46)	25 (8 - 51)	0.254
210	11 (-6 - 35)	17 (0 - 37)	0.470
240	16 (-6 - 30)	8 (-5 - 27)	0.709
270	6 (-14 - 17)	6 (-7 - 21)	0.829
300	9 (-21 - 25)	0 (-11 - 15)	0.755

ΔPI - delta of perfusion index.

* statistically significant p value for septic shock *versus* control. Perfusion index was measured in fingertip microcirculation following forearm stagnant ischemia. Values are expressed as medians and interquartile ranges (parentheses).

Table 3 shows the time to reach the peak of PI, the peak of PI (∆PI peak), and the mean of ∆PI measured at the first-minute interval (ΔPI_0-60_) and at the second-minute interval (ΔPI_60-120_) after deflation of the sphygmomanometer cuff. The peaks of PI were similar between the groups, although the peak was reached more slowly in the septic group. In addition, the reactive hyperemia was lower in the septic group in the early/mechanosensitive phase, as represented by ΔPI_0-60_. However, there were no statistically significant differences in the reactive hyperemia between the groups in the latter/metabolic phase, as represented by ΔPI_60-120_.

**Table 3:** Parameters of reactive hyperemia using the perfusion index in controls and patients with septic shock

Parameters	Control n = 35	Septic shock n = 47	p value
Time to peak PI (seconds)	48 (36 - 60)	70 (53 - 92)	< 0.001*
ΔPI peak (%)	79 (30 - 137)	71 (32 - 125)	0.853
ΔPI_0-60_ (%)	39 (6 - 75)	01 (-19 - 40)	0.001*
ΔPI_60-120_ (%)	48 (18 - 98)	43 (18 - 93)	0.586

PI - perfusion index; ΔPI peak - peak of perfusion index; ΔPI - delta of perfusion index.

* statistically significant p value for septic shock *versus* control.

With regard to correlation analysis (Table 4), the organ dysfunctions assessed by the SOFA score were only weak and positively correlated with the time-to-peak PI. In addition, there was a positive correlation between the ΔPI peak and vasopressor doses; there was a negative correlation between time-to-peak PI and C-reactive protein; there was a positive correlation between arterial pressure and (ΔPI_0-60_); and there were positive correlations between C-reactive protein, vasopressor doses, and ΔPI_60-120_. There were no statistically significant correlations with regard to the other parameters.

**Table 4:** Correlation tests between the time to peak perfusion index, delta peak of perfusion index, the early/mechanosensitive phase of reactive hyperemia, the latter/metabolic phase of reactive hyperemia and clinical-hemodynamic parameters

Parameters	Correlation analysis (r)							
	**ΔPI peak**	**p value**	**Time to peak**	**p value**	**ΔPI0-60**	**p value**	**ΔPI60-120**	**p value**
SOFA score	0.08	0.55	0.29	0.04*	-0.10	0.47	0.11	0.45
Arterial lactate	-0.01	0.91	0.13	0.37	-0.18	0.21	-0.02	0.85
ScvO_2_	-0.26	0.10	0.05	0.76	-0.16	0.32	-0.21	0.19
Pv-aCO_2_	0.19	0.36	-0.02	0.88	0.17	0.40	0.04	0.85
MAP	0.04	0.79	-0.22	0.14	0.34	0.02*	0.04	0.74
C-reactive protein	0.31	0.06	-0.34	0.03*	0.26	0.13	0.34	0.04*
Noradreline dose	0.41	0.004*	-0.04	0.74	0.23	0.13	0.42	0.003*

ΔPI peak - peak of perfusion index; ΔPI - delta of perfusion index; SOFA - Sequential Organ Failure Assessment; ScvO_2_ - central venous oxygen saturation; Pv-aCO_2_ - venous to arterial carbon dioxide difference; MAP - mean arterial pressure.

* statistically significant p value for Spearman correlation tests.

## DISCUSSION

This is the first report to assess the time-response analysis of reactive hyperemia in septic shock evaluated with the PI. The main finding of this study is that septic shock, after fluid resuscitation, leads to peripheral hypoperfusion associated with early hyporesponsiveness and considerably preserved vascular reactivity in the posterior phase. Thereby, it is possible to suggest the existence of a time-dependent peripheral hyperemic response in septic shock with a significantly microvascular ischemic reserve.

Multi-organ microcirculatory disturbances in sepsis, when not corrected, seem to exert a robust influence on outcomes.^(^^2^^,^^3^^)^ One commonly found microcirculatory disturbance is impaired vascular reactivity,^(^^5^^)^ and it can be estimated with reactive hyperemia, which is the increase in organ blood flow following brief arterial occlusion. Its most common clinical use is the evaluation of endothelial function through the NO bioavailability of conductance vessels.^(^^22^^)^ Another potential clinical use consists of analyzing the global tissue microvascular reactivity to represent the proportion of recruitable capillaries, arterioles and small arteries upon minimal flow delivery.^(^^4^^)^

Previous studies have shown that reactive hyperemia was clearly reduced in septic shock when evaluated in NO-dependent conduit arteries, demonstrating endothelial damage as an early marker of unfavorable prognosis.^(^^6^^)^ Additionally, in skeletal muscle microcirculation, impaired reactive hyperemia was related to organ failure, suggesting a link between abnormal microvascular ischemic response and tissue hypoperfusion.^(^^4^^)^ Inversely, our results demonstrated that reactive hyperemia, when measured in the fingertip with the PI, has a time-dependent dynamic in septic shock; it seems to be clearly reduced only in the first 45 seconds after ischemic stimulus and remains largely preserved after this period. In addition, the peaks of reactive hyperemia were similar, although the peak was reached more slowly in the septic shock group.

On first view, these results could suggest that the peripheral microcirculation could be relatively spared from the severe vascular damages that occur in septic shock. However, previous reports consistently have shown, in animals^(^^23^^)^ and humans,^(^^24^^)^ that the classic immune/inflammatory alterations of sepsis also impact the skin microvasculature and adjacent tissues.

A possible explanation for these apparently contradictory results may be associated with the fact that reactive hyperemia magnitude depends on the organ,^(^^4^^,^^12^^)^ vessel type^(^^4^^,^^6^^)^ and hyperemic phase evaluated.^(^^21^^)^ Therefore, the magnitude also depends on the different metabolic pathways present in different tissues. While endothelial-derived NO strongly mediates conduit vessel response,^(^^6^^,^^22^^)^ and cyclooxygenase influences muscle reactive hyperemia,^(^^25^^)^ the reactive hyperemia of skin and adjacent tissues is mediated by other mechanisms, including sensory nerves and hyperpolarizing factors.^(^^12^^)^ Curiously, many mediators that act as hyperpolarizing factors in microcirculation are increased in human sepsis and include neuro-mediators such as calcitonin gene-related peptide^(^^26^^)^ and oxidative stress-derived hydrogen peroxide.^(^^27^^,^^28^^)^ Elevated levels of hyperpolarizing factors could explain the relatively preserved reactivity of skin microcirculation despite the damages of septic shock. An evaluation of these mediators and peripheral reactive hyperemia is needed to confirm this hypothesis.

The phase of hyperemic response is also important because early flow responses seem to be mainly derived by mechanosensitive mechanisms, while shear-stress and metabolic factors affect late flow responses.^(^^21^^)^ These facts could explain the previous diverse results measured with near-infrared spectroscopy in sepsis (NIRS) that evaluated only the initial seconds of the first hyperemic phase.^(^^4^^)^ The evaluation of the initial hyperemic phase in septic shock using PI (ΔPI_0-60_) corroborates the findings of the results using the NIRS method and presumably adds new information about the latter hyperemic phases.

Our results are also in line with those of Engelberger et al.,^(^^29^^)^ who demonstrated that acute endotoxemia in humans, an acute phase sepsis model, selectively inhibits the NO-dependent vascular reactivity of human skin, while the post-ischemic reactive hyperemia remains preserved.

Interestingly, the systemic inflammation inferred by levels of C-reactive protein also seems to have a different influence on reactive hyperemia measured with the PI. While other reports showed a clear negative correlation with measurements in conduit vessels,^(^^30^^)^ our results showed a weak-to-moderate positive correlation at the metabolic phase of reactive hyperemia (ΔPI_60-120_), which also suggests an inverse response pattern in these vascular territories.

With regard to adrenergic response, our results showed a positive correlation between noradrenaline doses, the peak of reactive hyperemia and the metabolic phase of reactive hyperemia (ΔPI_60-120_). In addition, as expected, we showed reduced peripheral perfusion in septic shock patients compared to controls. It is well known that an intense redistribution of blood flow from non-vital organs to vital organs characteristically occurs in states of shock, causing peripheral hypoperfusion.^(^^31^^)^ The peripheral hypoperfusion is generated by an increase in sympathetic-neurohumoral activity^(^^31^^,^^32^^)^ and is associated with worse systemic perfusion and poor prognosis.^(^^15^^,^^32^^)^ Since previous reports showed that the PI is a very sensitive method for assessing adrenergic responses,^(^^33^^,^^34^^)^ the adrenergic stimulus (secondary to the sympathetic response to shock and to use of vasopressors) also becomes a direct hypothesis that could explain the relatively preserved peripheral vascular reactivity despite the peripheral hypoperfusion in the septic group: it implies the existence of a peripheral ischemic reserve in septic shock.

Nevertheless, previous clinical reports consistently have shown that reactive hyperemia tends to be reduced, not increased, by direct α-adrenergic stimulus when measured in cutaneous circulation.^(^^35^^,^^36^^)^ Similar results occurred in conductance vessels^(^^37^^)^ and muscle sympathetic nerves.^(^^38^^)^ Even in the first minute after deflation, where reactive hyperemia was reduced in the septic shock group (ΔPI_0-60_), there was no correlation with noradrenaline doses. Another interesting fact is that sepsis impairs adrenergic signaling and generates receptor hyporesponsiveness.^(^^5^^)^ Thus, the requirement for increasing doses of vasopressors could reflect adrenergic desensitization. Additionally, these results are not adequate to differentiate the sympathetic response to shock and the direct adrenergic effects of vasopressors. Thus, the precise role of sympathetic stimulus in the hyperemic response of the PI needs to be elucidated further.

Independent of the pathophysiologic cause, our results suggest that the presence of the early hyporesponsiveness or the non-utilization of the microvascular ischemic reserve does not seem to be associated with systemic anaerobic metabolism in septic shock after fluid resuscitation because there was not a correlation between reactive hyperemia (initial and later phases), ScvO_2_ and blood lactate. It is worth noting that this occurs despite the positive correlation of the early phase of reactive hyperemia and arterial pressure. These results are compatible with the previously described uncoupling between macrocirculation and microcirculation in septic shock.^(^^39^^)^

Finally, we found a weak but positive correlation between organ failure measured with SOFA score and time to peak of PI. Contrary to the report by He et al.,^(^^16^^)^ we did not see any correlation between the SOFA score and the peak of reactive hyperemia measured with PI. In addition, their report found higher values of reactive hyperemia in healthy subjects compared to those in the septic group, probably due to methodological differences between the studies. As previously described by our group, older patients present increased basal PI values and lower reactive hyperemia; further, cardiovascular risk factors such as hypertension, diabetes mellitus and smoking can influence the reactivity test.^(^^17^^)^ Thus, these factors were controlled in our study.

This study had limitations. First, this was a monocentric preliminary study with a high mortality rate (tertiary hospital). We acknowledge that this sample was relatively limited and that some of our findings should be interpreted as generating hypotheses. Thus, a bigger multicenter study, with a sample size calculation, is needed to confirm these findings. Second, a single measure was obtained, thus limiting our conclusions about intra-individual reproducibility. A temporal evolution study is needed to verify the potential of ΔPI as a dynamic parameter. Third, the number of patients did not allow to perform an outcomes analysis in a prospective study. A study by our group is underway to verify these aspects. Finally, one can argue that fingertip skin microcirculation is not representative of vital organ microcirculation. However, monitoring non-vital vascular beds has received increasing attention because these vascular beds are among the first to deteriorate and the last to be restored after resuscitation in common scenarios of shock and prediction outcome.^(^^10^^,^^11^^)^ Therefore, these results add important information to clinical monitoring because they show the dynamic response of peripheral reactive hyperemia in septic shock and suggest that peripheral hypoperfusion is more functional and less structural than thought previously.

## CONCLUSIONS

In conclusion, in this preliminary study, we found that reactive hyperemia in septic shock patients, when evaluated with the perfusion index, appears to be impaired only in the early phase of the post-ischemic response and remains considerably preserved in the latter phase, despite the severe vascular damages of sepsis. The reactive hyperemia evaluation with the perfusion index does not seem to be correlated with systemic macro-hemodynamics, and only the time-to-peak perfusion index is weakly correlated with organ failure, as assessed by the SOFA score. We hypothesize that the mechanisms responsible for these findings could be mediated by the sympathetic activity or immunometabolic mediators. However, further investigations are necessary to clarify these assumptions.
